# Optimization of bioremediation-cocktail for application in the eco-recovery of crude oil polluted soil

**DOI:** 10.12688/aasopenres.13028.1

**Published:** 2020-04-22

**Authors:** Joseph E. Agbaji, Eucharia O. Nwaichi, Gideon O. Abu

**Affiliations:** 1 Institute of Natural Resources, Environment, and Sustainable Development (INRES), University of Port Harcourt, Port Harcourt, Nigeria; 2 Department of Biochemistry, Faculty of Science, University of Port Harcourt, Port Harcourt, Rivers, 2340, Nigeria; 3 Department of Microbiology, Faculty of Science, University of Port Harcourt, Port Harcourt, 2340, Nigeria

**Keywords:** Bioremediation, Cocktail, Rhizobacteria, Response Surface Methodology, Agrowaste residues, Pollutants, Environmental modelling

## Abstract

**Background**: Environmental sustainability is the driver for finding the optimal bioremediation cocktail with the combination of highly potent hydrocarbonoclastic strains and the nutrient additives that significantly enhance mineralization of crude oil in polluted soil in order to mitigate its deleterious effects on the environment. In this study, four hydrocarbon-degrading bacterial strains were pre-selected from mined rhizobacterial isolates in aged crude oil-contaminated soil.

** Method**: Agrowaste residues of poultry-droppings, corn chaff, and plantain peel were selected among others for their ability to support high biomass of selected bacterial strains. Baseline proximate analysis was performed on the agrowaste residues. Simplified, one variable at a time (OVAT) was employed in the validation of the variables for optimization using the Multivariate analysis tool of Response Surface Methodology (RSM). To test the significant formulation variables, the Box-Behnken approach using 15 runs design was adopted.

**Results**:  The rate of contaminant removal was observed to fit into a quadratic function. For optimal rate or contaminant removal, the fitted model predicted the optimal formulation cocktail condition to be within 0.54 mg/kg (Corn steep liquor), phosphate 137.49 mg/kg (poultry droppings) and 6.4% inocula for initial TPH of 9744 mg kg ^-1^ and THC of 9641 mg kg ^-1^ contaminant level. The model for the application of the bioremediation product and the variables evaluated had a significant p-value < 0.005 for the attainment of 85 to 96 % of TPH and THC removal after 56 days of treatment.

**Conclusions**:  This study has shown the need to harness the abundant agrowaste nutrients in supporting high throughput rhizobacteria in the formulation of a bioremediation agent suitable for use in the reclamation of oil spill sites in the Niger Delta oil-producing region.

## Introduction

Pollution has remained a global threat, including in the Oil-rich Niger Delta Region, Nigeria ( Ite *et al.*, 2013). Society is now awash with chemical and physical remedial options. Modifications and innovations of many unsustainable remedial treatments have been reported in both local and international peer-reviewed journals ( Das & Chandran, 2011; Nwogu *et al.*, 2015). The Niger Delta has remained a recurrent site for oil spilling resulting from exploration and exploitation activities for crude oil ( Kadafa, 2012). Oil bunkering, sabotage, and poverty have been identified as key factors contributing to the increase in the cases of oil spills. Oil bunkering as it relates to Nigeria, refers to the illegal or the criminal act of breaking into pipeline carrying crude oil for the purpose of diverting the crude oil for sale to international oil theft syndicates or to local artisanal refinery for the sale of its refined products ( Nwilo & Badejo, 2006). The oil producing communities of the Niger Delta have witnessed a steady decline in their livelihood due to the devastating effect of oil exploration and exploitation activities on their environment and as such could not engage productivity in the economic activities of farming, fishing, and hunting ( Uyigue & Agho, 2011). This is further worsened by the infrastructural neglect from the oil companies and the central government who collect all oil revenue, leading to communities protesting against these companies and the central government by way of sabotage or vandalization of crude oil pipeline resulting in oil spillage ( Ogbeibu & Uyigue, 2015).

According to Dhote *et al.* (2017), the components of these crude oil pollutants and contaminants could contain mutagens and carcinogens, and their presence in an environmental media can lead to a flux or incremental change in the native microbial community structure and their function ( Varjani & Upasani, 2017). The shortage of limiting nutrients such as nitrogen and phosphorus occasioned by the large presence of carbon from the oil spill is one major factor affecting the metabolism of hydrocarbon by indigenous flora ( Kirkpatrick *et al.*, 2008). Hydrocarbon fractions such as total petroleum hydrocarbons have been reported to have both persistent and recalcitrant effects in the environment, leading to most of the health issues reported to be associated with crude oil pollutions ( Al-hawash *et al.*, 2018; Saggar, 2011). Leaks, facility-failures, accidents, and sabotage by host communities have taken centre stage in public discussion on cause and treatment of the impacted environment ( Das & Chandran, 2011). Corrective measures have involved a number of biological, chemical and physical approaches. The fusion of white and green technology in contaminant removal may provide a level of sustainability in the development of clean-up processes ( Abu, 2017).

Bioremediation is a process based on the application of biodegradation science ( Abu, 2017). It has been redefined to mean the use of microbial life and their products in the modification or removal of any pollutant or treatment of an environmental problem using biological processes ( Cohen *et al.*, 2004), having been applied in petroleum and oil field chemical units due to its ability to convert pollutants into less harmful forms ( Hussain *et al.*, 2009). It is regarded as cost-effective ( Hussain *et al.*, 2009), eco-friendly, feasible, sustainable and less invasive compared to physical and chemical methods ( Bramley-Alves *et al.*, 2014). The changes in the physicochemical properties of the environmental media have been associated with the severity of crude oil-related pollutants and the toxicity they exert to the indigenous flora ( Nwilo & Badejo, 2006). This may be linked to the removal of limiting nutrients ( Gaskin & Bentham, 2010). It could be *In-situ* or *Ex-situ* depending on the site of treatment ( Abu, 2017). Intrinsic features, microbial qualities and geotechnical indices of soil could enhance or hamper the quality of outcome ( Vidali, 2001). The bioremediation-cocktail is a mixture of bacterial consortia and nutrients designed for application in waste or pollutant remediation. The cocktail formulation mimics biostimulation (nutrient addition) and bioaugmentation (introduction of native microorganisms) for the eco-recovery of an impacted matrix. The cocktail nutrient was formulated in tandem with reported ratio of 100:10:1:0.5 for C:N:P:K for an enhanced microbial activity to achieve effective biodegradation of hydrocarbon pollutants ( Peekate & Abu, 2017). Cocktails could be designed as cost-effective materials for the treatment of waste of any kind.

Response surface methodology (RSM) is a multivariate statistical technique used in optimization of operational or experimental variables for multiple response analyses. This tool was first proposed by Roquemore, in 1976. It has been described by several peer-review articles as being a multi-disciplinary tool for robust analysis ( Pi *et al.*, 2016). Software programs based on the response surface methodology such as MODDE, Design Expert (DX), Minitab, XLSTAT add-ins, and GMP have been written to widen the application of this statistical technique. According to Pi *et al.* (2016) application of RSM is useful in biodegradation of crude fractions such as phenols, alkanes, and cyclic compounds. The Design Expert program includes several symmetrical experimental designs such as the Central Composite, Plackett-Burman, and Box-Behnken which are considered useful in optimization studies. Furthermore ( Zhang *et al.*, 2011) reported that the Design Expert software has been applied in certain enzyme catalysis, bacterial growth, and process design. It has the capacity to reduce processing time and the number of experimental test runs; can easily converge on the most desirable combination, or sweet spot; and could optimize parameter attributes for formulated product response.

## Methods

### Microorganism

Microorganisms used in this study, *Achromobacter agilis*, *Pseudomonas fluorescens*, *Bacillus thuringiensis*, *Staphylococcus lentus* were sourced from rhizobacterial flora of weeds harvested from aged crude oil impacted soil in Bodo, Gokana, Ogoni land of Rivers state, Nigeria (36° 4’N and 15° 7’E). The strains were isolated in the environmental biotechnology laboratory of the Department of Microbiology of the University of Port Harcourt, Rivers State, Nigeria. The total bacterial population in the oil-polluted rhizosphere soil sample was enumerated and isolated adopting serial dilution and the standard plate count technique using the pour plate method ( Ajayi & Abiola, 2018). Ten grams of the soil sample was measured into a conical flask and 90ml of sterile normal saline was mixed with the sample. The suspension was subjected to a shaker for three hours to homogenize the solution and this served as the stock solution. Ten-fold serial dilution of all the homogenized mixture was carried out using sterile normal saline as diluents. Seven test tubes containing 9ml of normal saline were used for the serial dilution. Aliquots of 1ml from 10 ^-5^ and 10 ^-7^ dilutions were introduced into duplicate sterile petri dishes and 20ml of molten nutrient agar incorporated with nystatin (N6261, Sigma-Aldrich) to suppress fungal growth was poured into the plates and swirled to allow homogenization. The plates were incubated at 37°C for 24 hours after which colonies on the plates were enumerated and subculturing of bacterial isolates was done to obtain a pure culture. Bacterial colonies were picked with a sterile inoculating loop and streaked on freshly prepared nutrient agar plates ( Olukunle, 2013). The plates were incubated at 37 °C for 24 hrs. Aliquots of 1ml from dilutions of 10 ^-5^ and 10 ^-7^ were also plated in duplicates on Bushnell Haas Agar (Lab M, China), using the spread plate technique; 100 µgml ^-1^ Nystatin (N6261, Sigma-Aldrich) were added to the Bushnell Haas Agar (Lab M, China) to suppress fungal growth. A filter paper saturated with sterile crude oil was aseptically placed on the inside of the inverted Petri dishes and the culture plates were incubated for 14 days at 37°C. Plates containing colonies were afterwards enumerated for the bacterial load ( Ajayi & Abiola, 2018).

### *In-Vitro* screening for hydrocarbon degradation potential of bacterial isolates

Crude oil biodegradation screening of the organism was determined by the method given by Shekhar *et al.* (2015). In vitro hydrocarbon degradation potential was determined using 18h old bacterial inoculum (1ml) transferred into 98ml Bushnell-Hass medium (Lab M, China) at pH 7.0 and was supplemented with 1ml of Bonny light crude oil as the carbon source. It was incubated at 37°C at 170 rpm in shaker incubator for a period of 7days. The hydrocarbon degradative potential of the bacterial isolates was both monitored by viable plate count and optical density (OD) method. The growth of the bacterium was measured by taking the optical density (OD) readings at 600nm for 7 days at regular 1-day intervals by a spectrophotometer, against the Bushnell Haas medium as blank. A corresponding 5ml aliquot sample was collected for 1ml serial dilution viable plate count and a corresponding daily reading of pH was taken and recorded. All experiments were performed in duplicate ( Olukunle *et al.*, 2015; Dilmi *et al.*, 2017). The total viable plate count versus incubation time for each bacterial isolates were plotted into growth model curve, and using the first-order growth rate equation, the growth kinetic parameters such as specific growth rate were calculated. The viable plate count and optical density results were also analysed using one-way ANOVA. Both the specific growth rate and ANOVA results were the basis for the selection of the four isolates for the cocktail formulation.

### One Variable at Time (OVAT) Studies: Substrate selection and concentration range finding

A selected number of agrowaste residues of carbon, nitrogen, phosphorus and potassium source were used as an amendment with other mineral components for the mass cultivation of the selected rhizobacterial to monitor their growth pattern for optimization study. Mineral salt media (Na _2_ CO _3_ 3.0g, K _2_H _2_PO _4_ 3.0g, MgSO _4_. 7H _2_O 7.0g, NaCl 0.1g, Urea 4.0g, CaCl _2_. 2H _2_O 0.1g, Trace element 1ml) was compounded, prepared and fortified with Carbon (Guinea corn chaff, Corn chaff, Millet Chaff), nitrogen (Guinea corn liquor, Corn liquor, Millet liquor, Cow blood meal, Cow urine), phosphates (poultry dripping, Bone Char, Crab Char), potassium (Plantain peels, Wood ash) sources. An aliquot of 1% of bacterial inoculum was seeded into the media. The set up was incubated at 37°C and 1.0 ml of the sample was obtained from the experimental setup at a 24h interval and then subjected to growth monitoring by viable plate count on nutrient agar and optical density at 600 nm. Range finding of nutrient for mass cultivation was developed for carbon source (Corn chaff) (0.0 gL ^-1^, 5.0 gL ^-1^, 10.0 gL ^-1^, 15.0 gL ^-1^, 20.0 gL ^-1^and 25.0 gL ^-1^), Nitrate (corn steep liquor) (0%, 10%, 20%, 30%, 40% and 50% (v/v)), Phosphorus (poultry droppings) (0.0 gL ^-1^, 0.5 gL ^-1^, 1.0 gL ^-1^, 1.5 gL ^-1^, 2.0 gL ^-1^ and 2.5 gL ^-1^) and Potassium (plantain peels)) (0.0 gL ^-1^, 0.05 gL ^-1^ 0.10 gL ^-1^, 0.15 gL ^-1^, 0.20 gL ^-1^ and 0.25 gL ^-1^) ( Hanif *et al.*, 2018; Nwaichi & Wegwu, 2012; Peekate & Abu, 2017). The results were analysed using two-way ANOVA in the selection of optimal nutrient source for optimization and the exponential phase from the bacterial growth data was used to deduce point of first-order kinetics. The substrate-bacterial growth dynamic was fitted into the Monod model and the optimal growth parameters was selected for and used in the bioremediation cocktail formulation and application study.

### Optimization of biococktail conditions for high throughput of bacterial strains

To optimise the bacterial yield and bioremediation efficiency, the carbon: nitrogen ratio, carbon: phosphorus ratio, potassium (biochar) (w/v) and inoculum size were considered as independent variables while the bioremediation indices were applied as the dependent variable. The RSM program experimental design of Box and Behnken was employed ( Emeko *et al.*, 2015) to determine the optimum concentration of the significant independent variables and their mutual interactions effects on TPH and THC removal from the treated experimental samples. Each independent variable was assigned three different levels of concentration (low, medium and high, which are coded as –1, 0 and +1, respectively) with the experimental design centre point replicated three times for the estimation of error. The RSM program by Design-Expert version 11.0 product of Stat-Ease Inc. Minneapolis, USA was used for the experimental design and data analysis. The inputted experimental data was accorded a second-order polynomial regression model equation as the equation with the goodness of best fit and the dependent variables response were defined in terms of the independent variables as:

$$\text{Y}={\text{β}}_{\text{0}}+{\text{β}}_{\text{i}}\text{A}+{\text{β}}_{\text{j}}\text{B}+{\text{β}}_{\text{k}}\text{C}+{\text{β}}_{\text{ii}}{\text{A}}^{\text{2}}+{\text{β}}_{\text{jj}}{\text{B}}^{\text{2}}+{\text{β}}_{\text{kk}}{\text{C}}^{\text{2}}+{\text{β}}_{\text{ij}}\text{AB}+{\text{β}}_{\text{ik}}\text{AC}+{\text{β}}_{\text{jk}}\text{BC}\;(1)$$

Where Y is the predicted response (loss or removal of TPH and THC)

A: Nitrogen source, B: Phosphorus source C: inoculum size

β _0_: Intercept, β _i_, β _j_, and β _k_ are the linear coefficients, β _ii_, β _jj_, and β _kk_ are the squared coefficients, β _ij_, β _ik_, and β _jk_ are the interaction coefficients, A ^2^, B ^2^, C ^2^, AB, AC, and BC are the interactions between the variables as significant terms.

### Open access alternative

The study analysis was done with Design expert software and can be performed by any alternative or similar open access software like R-studio (R version 3.1.2 with installed rsm Package for surface and contour plots) which is open source and free for non-commercial purposes. Any Windows 7 and 64 bit upward with Windows graphics package can support the running of the Design expert software. RStudio Server version gives access to the RStudio IDE (integrated development environment) from anywhere via a web browser, debugs in an interactive manner and runs on the desktop (Windows, Mac, and Linux) or in a browser connected to RStudio Server with boot, class and cluster as recommended packages. Readers and reviewers can replicate this analysis using a detailed unrestricted access methodology described in Chapter 10 of Wu & Hamada (2009). It is recommended that readers look at YouTube tutorial videos on design and optimization of experiments and practice with existing data in previous studies to verify result with ones in those articles.

The design of the experiment for the formulation of the bioremediation cocktail followed three key steps of screening, characteristisation and optimisation (SCO). The combination of knowledge of subject matter and One Variable at a Time (OVAT) or One Factor at a Time (OFAT), was utilized in the screening and characteristion phases to narrow down to the vital few variables or factors necessary for the development of the bioremediation cocktail. In this case, four key variables were identified or selected as vital ingredients for the cocktail mixture. Consortia of four high through-put hydrocarbonoclastic rhizobacterial, two limiting nutrient sources (N-P) from corn steep liquor and poultry dropping, and a third non limiting but vital nutrient of plantain peels char was screened, selected and characterized to established their minimum and maximum concentration range at laboratory scale.

Optimization phase is the cocktail formulation phase and involves finding the vital factors or variables with their minimum and maximum range in concentration or amount. These pre-optimized variables are keyed into the variable view as given in choice software and the runs become the basis for the formulation of sets of unique cocktail mixtures, which are then applied to the same polluted sample soil size and the response reading for Total Hydrocarbon Content (THC) and Total Petroleum Hydrocarbon (TPH) are collected at certain time interval (days). The collected response or results of THC and TPH are re-input back into the software alongside its designed conditions, and the software generates a unique model, usually a quadratic model, where the response (THC or THP removal) is a function of the inputted variable. These software allow researchers or stakeholders to see the variables or factors that makes the greatest impact or effects on TPH or THC removal, the interaction of variables or factor and their effects on THC or TPH removal, and the power factors effects of the variables or factors on THC or THP removal.

### Bioremediation studies

Two-kilogram (2.0kg) of soil was spiked with 15 cl of Bonny Light crude oil sourced from the Nigerian National Oil Corporation (NNPC) refinery at Port Harcourt, resulting to a Total Hydrocarbon Content (THC) of 9744 mg/kg soil where each distributed into 15 pre-perforated earthen pots. These were treated with nutrients and microbial-designed cocktail mixture in ratios described in Table 2 as modelled by the RSM-DX program. Also, a non-treated sample was set up as control. The experimental setup was carried out in the open greenhouse of the Department of Plant Science and Biotechnology, University of Port Harcourt. Samples were collected from the setup at 7-day intervals after stirring and then sparged with water daily.

### Determination of total hydrocarbon content (THC)

The calibration and reference standard curve began with the preparation of a standard gradient of the Bonny light crude oil in triplicate, each dilution was analysed on a UV-vis spectrophotometer (Agilent, Cary 55B) to obtain the different absorbance (A) signals vs wavelength (λ) of 450 nm. The data was regressed on an absorbance vs concentration graph and the variables were calculated from the coefficient of the line graph. Soil sample of 5g was weighed into the brown extraction container and conc. sodium sulphate (239313, Sigma-Aldrich) was added to remove water. Then Di-chloromethane (1.00668, Supelco) was added to extract the hydrocarbon content that was in the soil sample. The solution was then filtered through a funnel packed with cotton wool impregnated with sodium sulphate and silica gel (1.01907, Supelco). The resultant filtrate was then analysed using the UV-vis spectrophotometer at a wavelength of 450nm. This was used to read off the corresponding THC concentration from the standard curve.

### Determination of total petroleum hydrocarbon (TPH)

The soil sample (5g) was poured into a 1-litre separatory funnel from the glass sample bottle where it was preserved. 50ml of methylene chloride (1.00668, Supelco) was transferred to the sample bottle, sealed, and vortexed for 30 seconds to purge the inner surface of grease. The entire mixture was transferred to the separating column containing the soil sample and while the supernatant was collected by vibrating the separation column for two minutes with intermittent venting to allow excess pressure to escape. The organic phase was allowed to separate from the water phase for about ten minutes, then the methylene chloride extract was collected in a 250ml Erlenmeyer flask. 60ml of methylene chloride was distributed into the sample container to purge the container of grease and the column with 20ml of the methylene chloride into the supernatant collected. The protocol for extraction was repeated the second and third time. The combined supernatant was collected into an Erlenmeyer flask. This was followed by drying the supernatant through a separation column packed with cotton wool saturated with anhydrous sodium sulphate and silica gel. The collected supernatant was transferred into the flask and was concentrated by blowing it down with nitrogen gas to 1.0ml. The remaining extract was mixed with 1.0ml of the methylene chloride and 1.0µl was injected into the gas chromatograph (Agilent, 6890 Series) fitted with a flame ionization detector for TPH analysis.

### Proximate analysis and mineral content determination

Proximate analysis is a quantitative analysis used to determine the different macro and micro nutrients components in organic material ( Ekwuabu *et al.*, 2016). In this study. mineral nutrient content of phosphate, nitrogen, and potassium was determined by ascorbic, Kjeldahl and the Atomic Absorption Spectroscopy‎ (AAS) methods respectively. While the total organic carbon (TOC) was also determined in each compound according to ASTM, 2000 Ekwuabu *et al.* (2016). The agrowaste material being analysed are corn chaff for TOC, corn steep liquor for Nitrate-Nitrogen, poultry droppings for phosphorus content, and plantain peels for potassium. Corn (IT 45) and Plantain (IT 09) were sourced from International Institute of Tropical Agriculture (IITA) while poultry droppings were freshly ssourced from Emmanuel farms Aluu Rivers state Nigeria. The reported results were TOC of 99.5 % for corn chaff, 3.31 mg L ^-1^ of nitrate-nitrogen for corn steep liquor, 2.42% of phosphorus content for poultry droppings and 176.04ppm of potassium content for plantain peels.

### Determination of Total Nitrogen (Kjeldahl Method)

Soil sample was weighed (0.1g) into a clean 250ml conical flask, 3g of digestion catalyst of copper sulphate (451657, Sigma-Aldrich) and sodium sulphate (239313, Sigma-Aldrich) was added and 20ml concentrated sulphuric acid (339741, Sigma-Aldrich) was also added and the sample was heated to digest with the content colour turning from black to sky-blue coloration. The content was cooled to room temperature and was diluted to 100ml with distilled water. 20ml of the diluted digest was put in a heated distillation flask attached to a Liebig condenser connected to a receiver containing 10mls of 2% boric acid (B0394, Sigma-Aldrich) indicator. 40ml of 40% sodium hydroxide (795429, Sigma-Aldrich) was injected into the digest via a syringe until the digest became strongly alkaline. The mixture was heated to boiling and distilled ammonia gas condensed into the beaker containing the boric acid, turning purple to greenish coloration. The distillate was titrated with standard 0.1N hydrochloric acid (258148, Sigma-Aldrich) solution changing the colour back to purple from greenish. The volume of hydrochloric acid added to affect this change was recorded as the titrate value

Calculation:

$$\text{\% organic nitrogen = }\frac{\text{titrate value × 1.4 × 100 × 100}}{\text{1000 × 20 × 0.1}}$$

Where titrate value = the volume of HCl used in titrating the ammonium distillate.

1.4 = Nitrogen equivalent to the normality of HCl used in the titration 0.1N.

100 = the total volume of digest dilution

100 = percentage factor

1000 = conversion factor from gram to milligram

20 = integral volume of digits analysed or distilled

0.1 = the weight of the sample in gram digested

### Determination of Total Organic Carbon (TOC)

Dried soil sample (0.1g) was weighed into a 250ml conical flask, 5ml of potassium dichromate (207802, Sigma-Aldrich) and 7.5ml concentrated sulphuric acid (339741, Sigma-Aldrich) was added to the mixture. A separate 250ml conical flask containing 5ml of potassium dichromate (K _2_Cr _2_O _7_) and 7.5ml concentrated sulphuric acid was prepared. The sample was heated for 15 mins after which they were allowed to cool to room temperature before diluting to 100ml with distilled water. 10ml diluted digest was measured into a separate 250ml conical flask and 2 drops of ferone or 4-Methylumbelliferone (M1381, Sigma-Aldrich) were added as an indicator, the sample was titrated with ferrous ammonium sulphate (215406, Sigma-Aldrich) until the colour changed to leafy green, at which point the titrate value was recorded.

Calculation:

$$\text{\% organic carbon = }\frac{\text{Blank titrate value – Sample titrate value × 0.2 × 0.3}}{\text{Weight of sample used}}$$

### Determination of Phosphate- Phosphorus (Ascorbic Method)

Soil sample (5g) was extracted with 50ml of 2.5% acetic acid (71251, Supelco). The extract was filtered into a 250ml conical flask. A blank and standard phosphate ion concentration (103935, Supelco) ranging from 0.0001 – 0.0007 was prepared and 0.8ml already prepared combined reagent of molybdate (69888, Supelco) and ascorbic acid (100468, Supelco) was added respectively. The bluish colour developed within a 30 mins interval and optical densitity was read at 840nm using a spectrophotometer (Thermospectronic, Genesys 20 series). Vital settings as recommended for the instrument employed were programmed. The extracted sample volume developed was also read at the same wavelength. The concentration of the phosphate ion in the sample was extrapolated from the standard graph plotted with the value from the standard phosphate ion range.

Phosphorus ion in the soil was obtained by multiplying the phosphate ion by factor of 0.3262 (factor of 0.3262 was derived from the ratio of molecular weight of the total phosphorus to the molecular weight of the total phosphate).

### Determination of Potassium (AAS Method)

Substrate sample (0.50g of oven dried) was weighted into a 100ml Kjeldahl flask. 1ml 60% perchloric acid (244252, Sigma-Aldrich), 5ml nitric acid conc. (258113, Sigma-Aldrich) and sulphuric acid conc. (339741, Sigma-Aldrich) was added. The mixture was swirled gently and digested (causing precipitation due to heat application) slowly at moderate heat for 10 to 15 minutes until the appearance of white fumes. The digest (precipitate) was set aside to cool. The digest was filtered (NO. 44 paper) into a 50ml volumetric flask and diluted to volume giving a concentration of roughly 1% (v/v). A blank digestion was carried out in the same way. Atomic Absorption Spectroscopy (Agilent 4200 series) was used to measure the atomic absorption of the potassium ion concentration in the sample, with the lamp was set to 776nm wavelength to take the reading. Slit width, air and gas pressure was adjusted. Other vital settings as recommended for the instrument employed were programmed. Standard potassium ion concentrations were aspirated into the instrument “burner chamber” to calibrate the equipment and to plot a graph of a standard calibration curve to determine ppm K in the sample solutions. The aspirator tubing’s system was occasionally flushed with water before samples were aspirated. The blank sample was carried out in the same way and subtracted where necessary.

Calculation:

$$\text{C (\%) = }\frac{\text{C(ppm) × solution volume (ml)}}{\begin{array}{l} \text{104 × sample wt. (g) (where 104 is the conversion factor}\\ \text{for the reproducibility for the determination of potassium)} \end{array}}$$

Where C = ppm (K) obtained from the graph

Apply factors for dilution or concentration and correct to dry weight where necessary

### Consideration and Deductions of Agrowaste Nutrient Concentration from Proximate Analysis Results

The following estimate analysis is to deduce the nutrients concentration from the agrowaste residues proximate analysis results. This is important for the experimental design of the bioremediation cocktail and for the reproducibility of the cocktail formulation.

### Estimation of Nitrate-Nitrogen (NO _3_-N) Concentration Value for Optimisation of Bioremediation Cocktail Model Design

Nitrate- nitrogen concentration in corn steep liquor = 3.31mgL ^-1^ = 0.00331mgml ^-1^

Bacterial-substrate growth and variance analysis of the OVAT experimental data for corn steep liquor estimated optimal nitrate substrate to range from 10 to 20ml of the corn steep liquor for optimal bacterial specific growth rate and enzyme activity. The experiment was set up in 100ml of growth media.

Scaling up the laboratory data to bioremediation setup, mean multiplying by a factor of 10 (100ml to 1000ml). Where 1000ml of water = 1kg = 1000g (2000g of soil used in remediation setup)

Therefore, corn steep liquor for bioremediation ranged from 100ml to 200ml.

Resulting in a Nitrate-Nitrogen (NO3-N) concentration of 0.331mg/100ml to 0.662mg/200ml for a kilogram of the sample soil treated in the bioremediation setup.

### Estimation of Phosphorus Concentration Value for Optimisation of Bioremediation Cocktail Model Design

Phosphate (PO _4_) concentration in poultry droppings = 2.42 % of the measured sample

While Phosphorus (P) concentration is estimated from the derived molecular equation

The molecular mass of PO _4_ = 95g/mol; The molecular mass of P = 31

Thus, % of phosphorus = 31/95= 33%

Bacterial-substrate growth and variance analysis of the OVAT experimental data for poultry droppings estimated optimal phosphorus substrate to range from 0.05 to 0.25g of the poultry droppings for optimal bacterial specific growth rate and enzyme activity. The experiment was set up in 100ml of growth media.

This value was scaled up to bioremediation setup by a factor of 100

Therefore, bioremediation cocktail values of 5g to 25g for a kg of the soil sample were used.

Phosphate (PO _4_) mass conc. in a 5g Poultry Droppings is = $\frac{\text{2.42 × 5}}{\text{100 × 1}}$ = 0.121g

Phosphorus (P) mass conc. = 0.121 × 0.3262 = 0.0394g = 39.47mg

Phosphate (PO _4_) mass conc. in a 25g Poultry Droppings is = $\frac{\text{2.42 × 25}}{\text{100 × 1}}$ = 0.605g

Phosphorus (P) mass conc. = 0.605 × 0.3262 = 0.197g = 197.35mg

Therefore, Phosphorus (P) mass concentration ranged from 39.4mg to 197.4 mg for kilogram of the sample soil treated in the bioremediation setup.

### Estimation of Potassium Concentration Value for Optimisation of Bioremediation Cocktail Model Design

Potassium was evaluated in the form of plantain peel which was charred and made into powder of similar size. Though, not a major nutrient for bacterial growth but has a role in microbial activities both as an enzyme activator and as the predominant monovalent cation for maintenance of cell turgor ( Kovárová-Kovar & Egli, 1998). Its char form acts as an absorption site for bacterial and substrate interaction in the remediation setup.

Bacterial-substrate growth and variance analysis of the OVAT experimental data for plantain peels estimated potassium substrate of 0.025g for optimal biomass and viable bacterial count of bacterial isolates. The experiment was set up in 100ml of growth media. This value was scaled up to bioremediation setup by a factor of 100

Therefore, a 2.5g of plantain peel char was added to all cocktail formulation used for the bioremediation application.

The plantain peels char was not a variable in the RSM-DX program for optimization.

The concentration of the potassium in the plantain peel char was estimated from the mass spectrophotometry measurement result.

1.0g of plantain peels char was suspended in a 50ml extraction solvent used for the measurement.

Resulting in a value of 176.037ppm (where ppm = mgL ^-1^ and L = 1000ml and is equivalent to 20g of extract plantain peels

Thus, K equivalent = 176.037mg/20g sample of plantain peels char or 8.8mgg ^-1^ plantain peels char.

Therefore, 2.5g × 8.8mgg ^-1^ = 22mg of plantain peels char for kilogram of the sample soil treated in the bioremediation setup

### Cocktail Formulation

The bioremediation cocktail was formulated to offer the benefits of bioaugmentation (consortia of four high throughputs hydrocarbon-degrading bacteria selected through hydrocarbon degradation screening study) and biostimulation (three agrowaste residues that are sources of N:P:K selected from OVAT nutrient and concentration range study).

The following bacterial isolates *Pseudomonas fluorescens, Achromobacter agilis, Bacillus thuringiensis, and Staphylococcus lentus* were selected for their high hydrocarbon-degrading capabilities and corn steep liquor for Nitrogen and poultry droppings for Phosphorus were selected for their ability to support mass cultivation of the selected isolates. However, plantain peel char powder was not selected just for its potassium content benefit to microbial activities, which experimental OVAT values both statistically and analytically indicate a constant nutrient value across all bacterial isolates. Plantain peel char was selected more for its biochar influence on bioremediation. As the only agrowaste residue charred, it was an ideal mix for the cocktail formulation.

The laboratory results were analysed using both ANOVA and the Monod model for bacterial growth dynamics to select the optimum nutrients concentration range and results were inputted into the RSM-DX program. The Random Surface Model has the following as the independent variable or factors; Nitrogen, Phosphorus and Inoculum Size. The result of the RSM-DX program was a randomization of nutrients and microbial consortia in ratios designed to formulate a bioremediation cocktail mixture (see Table 2 for cocktail ratio). Each ratio of the nutrient and microbial consortia is a unique portion of the biococktail and in each portion a 2.5g of plantain peels char is added to make the final cocktail mixture before it is applied to the experimental polluted soil sample.

### Experimental design

The experimental design was based on the randomized Box-Behnken complete block model with 15 remediation experimental runs, which includes three replicates and 12 unique treatments plus a control. The sample soil was sourced from the experimental location in Bodo, Gokana of Rivers State, Nigeria (36° 4’N and 15° 7’E), and geotechnical analysis was carried out. A sizable volume of the sample soil was sieved for uniform soil particles and spiked with Bonny light crude oil. A sample of the spiked soil was collected and run through a GC-FID for its TPH and THC values. This served as the reference value before the application of the bioremediation cocktail.

Soil spiked with Bonny light crude oil and weighting 2.0kg were distributed into 14 (13 randomized as modelled by the RSM-DX program and 1 control) pre-perforated open-air earthen pots. The pots were labelled 1 to 13 while the 14 ^th^ pot was labelled as the control. Similarly, pre-formulated and labelled 1 to 13 bioremediation cocktail as described from the RSM-DX program, were applied to their respective spiked soil samples.

The application was done such that Day zero was treated with 50% of the bioremediation cocktail after which soil sample was taken for TPH and THC measurement, 25% of the cocktail was applied after a sample was collected on Day 7 and the last 25% was applied on Day 14 after sample was collected for TPH and THC. The treated spike soil was thoroughly mixed or agitated after each application and sprinkle with water every day. Other samples were collected on Day 21 and Day 56 from the first day of treatment. The TPH and THC results were fed back to the RSM-DX program as actual results, which the model used to predict an optimized formulation for the bioremediation cocktail.

Input and output data from modelling as well as experimental data is provided as underlying data ( Nwaichi, 2020).

## Results

The results presented in Table 1 describe the initial and final THC and TPH values of the experimental samples during the treatment for pollutant removal. The control sample (S14) day zero value represents the initial THC (9641 mg kg-1) and TPH (9744 mg kg-1) values prior to treatment. All other Day zero samples values for both THC and TPH were taken after the application of the bioremediation cocktail. The treated samples' TPH measurement are all less than the control sample likely due to the biosurfactant leachate effect of the cocktail applied. However, for the purpose of modelling all day zero measurement were used as initial values.

**Table 1.** THC and TPH results of the experimental sample as measured by UV-Vis Spectrophotometer and GC-FID.

	Total Hydrocarbon Content (THC)					Total Petroleum Hydrocarbon (TPH)		
	Day 0	Day 7	Day 14	Day 21	Day 56	Day 0	Day 21	Day 56
Sample Order	mg/kg	mg/kg	mg/kg	mg/kg	mg/kg	mg/kg	mg/kg	mg/kg
S1	8626	7495	5927	3276	1193	8856	2926	1315
S2	8286	5573	2971	1579	629	8247	1794	498
S3	8423	6295	3682	1685	1251	8390	1960	641
S4	8214	4833	2160	1192	487	8193	1599	548
S5	8564	7159	5384	2903	1052	8639	2809	1105
S6	8240	5059	2564	1314	417	8236	1650	428
S7	8465	6521	4199	1928	619	8495	2007	604
S8	8187	4502	1984	1059	360	8157	1466	1167
S9	8515	7026	4921	2617	895	8523	2546	938
S10	8153	4341	1640	952	848	8098	1299	835
S11	8358	5906	3105	1606	724	8256	1879	762
S12	8489	6877	4511	2190	1065	8516	2179	986
S13	8074	4168	1397	890	268	8030	832	299
S14(control)	9641*	9448	9240	9038	8766	9744*	9134	8860

*The control sample (S14) day zero value represents the initial THC and TPH value prior to treatment. All other Day zero samples for both THC (Total Hydrocarbon content) and TPH (Total Petroleum Hydrocarbon) were taken after the application of the bioremediation cocktail. The treated samples' values are all less than the control sample likely due to the biosurfactant leachate effect of the cocktail. For the purpose of modelling all day zero value was treated as initial values*. *UV-Vis = Ultraviolet Visible and GC-FID = Gas Chromatography - Flame Ionization Detector.*

**Table 2.** Optimization of the Cocktail parameters for Optimum Degradation of Hydrocarbon using Box-Behnken Response Surface Model.

					21 Days Treatment		56 Days Treatment	
		Variable 1	Variable 2	Variable 3	Response 1	Response 2	Response 1	Response 2
Std Order	Run Order	A:Nitrate	B:Phosphate	C:Inoculum size	TPH Removal	THC	TPH Removal	THC
		mg/kg	mg/kg	%	%	%	%	%
5	1	0.331	118.41	5	66.96	62.02	85.15	86.16
6	2	0.662	118.41	5	78.24	80.95	93.96	92.41
11	3	0.4965	39.47	7	76.64	80.00	92.36	85.46
12	4	0.4965	197.35	7	80.48	85.49	93.32	94.08
1	5	0.331	39.47	6	67.48	66.10	87.21	87.72
8	6	0.662	118.41	7	79.97	84.05	94.81	94.93
2	7	0.662	39.47	6	76.38	77.22	92.89	92.69
3	8	0.331	197.35	6	82.03	87.07	85.75	95.61
10	9	0.4965	197.35	5	70.12	69.27	88.99	89.49
7	10	0.331	118.41	7	83.96	88.33	89.66	89.62
4	11	0.662	197.35	6	77.24	80.79	90.77	91.47
13	12	0.4965	118.41	6	89.64	88.97	96.28	96.69
14	13	0.4965	118.41	6	89.64	88.97	96.28	96.69
9	14	0.4965	39.47	5	74.41	74.20	88.48	87.48
15	15	0.4965	118.41	6	89.64	88.97	96.28	96.69

*THC = Total Hydrocarbon Content; TPH = Total Petroleum Hydrocarbon; Potassium (plantain peels char) was model in the cocktail ratio with a value of 22mg/kg*

The results of Table 2 describe the design matrix for the optimization of the bioremediation cocktail for responses on % THC and TPH removal at both 21st and 56th days of the monitoring. The experiment was designed with 15 runs. The order suggests an order for permutations of nutrients in the bioremediation cocktail. The centre points at runs 12, 13 and 15 had the highest bioremediation with 88.97 and 89.64% removal for 21 days monitoring and 96.69 and 96.28% removal for 56 days monitoring for THC and TPH respectively. The result presented in Table 3 shows the robust data analysis for the responses for being significant and the lack of fit for being non-significant, of the data tested, p-value 21st - 56th days of 0.031 – 0.028 and 0.009 – 0.002 was reported for THC and TPH removal rate respectively. Table 4 presents the model’s predictable values for THC and TPH removal at 21st and 56th days of monitoring using the experimental data as model input.

**Table 3.** ANOVA result for TPH and THC loss for the quadratic model.

	21 Days Cocktail Treatment						56 Days Cocktail Treatment					
Terms	TPH Removal			THC Removal			TPH Removal			THC Removal		
	F-value	p-value	Coefficient Estimate	F-value	p-value	Coefficient Estimate	F-value	p-value	Coefficient Estimate	F-value	p-value	Coefficient Estimate
Intercept			89.64			88.97			96.28			96.69
Model	10.87	0.009	significant	6.06	0.0307	significant	19.82	0.0021	significant	6.32	0.0281	significant
A-Nitrate	2.17	0.201	1.42	2.62	0.1666	2.44	68.73	0.0004	3.08	5.41	0.0676	1.55
B-Phosphate	3.74	0.111	1.87	4.33	0.0919	3.14	0.5058	0.5088	-0.2643	10.53	0.0228	2.16
C-Inoculum size	16.37	0.01	3.91	18.22	0.008	6.43	20.84	0.006	1.7	2.57	0.1698	1.07
AB	6.25	0.055	-3.42	4.17	0.0967	-4.35	0.0996	0.7651	-0.1659	5.84	0.0604	-2.28
AC	7.79	0.038	-3.82	7.41	0.0416	-5.8	3.04	0.1416	-0.9168	0.0609	0.8149	-0.2326
BC	2.21	0.198	2.03	1.5	0.2759	2.6	0.0451	0.8402	0.1116	3.07	0.14	1.65
A²	17.73	0.008	-5.99	4.67	0.0831	-4.79	41.16	0.0014	-3.51	2.59	0.1684	-1.58
B²	30.5	0.003	-7.86	8.3	0.0345	-6.39	43.69	0.0012	-3.62	10.88	0.0215	-3.24
C²	19.98	0.007	-6.36	5.81	0.0608	-5.35	11.77	0.0186	-1.88	19.44	0.007	-4.33

*THC = Total Hydrocarbon Content; TPH = Total Petroleum Hydrocarbon*

**Table 4.** The predicted and actual values for TPH and THC loss as determined by RSM.

		21 Days Cocktail Treatment				56 Days Cocktail Treatment			
		TPH Removal (%)		THC Removal (%)		TPH Removal (%)		THC Removal (%)	
Standard Order	Run Order	Actual Value	Predicted Value	Actual Value	Predicted Value	Actual Value	Predicted Value	Actual Value	Predicted Value
5	1	66.96	68.13	62.02	64.17	85.15	85.2	86.16	87.93
6	2	78.24	78.61	80.95	80.65	93.96	93.19	92.41	91.5
11	3	76.64	75.42	80	77.93	92.36	92.64	85.46	86.38
12	4	80.48	83.23	85.49	89.41	93.32	92.33	94.08	94.01
1	5	67.48	69.07	66.1	67.87	87.21	86.17	87.72	85.88
8	6	79.97	78.8	84.05	81.9	94.81	94.75	94.93	93.17
2	7	76.38	78.76	77.22	81.44	92.89	92.66	92.69	93.54
3	8	82.03	79.65	87.07	82.84	85.75	85.97	95.61	94.76
10	9	70.12	71.34	69.27	71.34	88.99	88.71	89.49	88.57
7	10	83.96	83.59	88.33	88.63	89.66	90.42	89.62	90.53
4	11	77.24	75.66	80.79	79.02	90.77	91.8	91.47	93.31
13	12	89.64	89.64	88.97	88.97	96.28	96.28	96.69	96.69
14	13	89.64	89.64	88.97	88.97	96.28	96.28	96.69	96.69
9	14	74.41	71.66	74.2	70.28	88.48	89.47	87.48	87.55
15	15	89.64	89.64	88.97	88.97	96.28	96.28	96.69	96.69

*THC = Total Hydrocarbon Content; TPH = Total Petroleum Hydrocarbon*

The values in Table 3 provides the statistical relevance of the RSM model results. The model F-value of 10.87 for 21 days treatment and 19.82 for the 56 days treatment implies the model is significant in terms of predicting TPH removal. Also, there is only a 0.86% and 0.21% probably chance that the obtained F-value from the 26 and 56 days model could occur due to noise. The model p-values are both less than the model reference p-value of 0.05 (5%) indicating that the model variable terms are significant in predicting the removal of TPH. The following variable terms of C, AC, A², B², and C² are significant variable terms in the 21 days model while the A, C, A², B², and C² are significant variable terms in the 56 days model for TPH removal. The other variable terms in both models are considered insignificant since their p-values are greater than 0.10 (10%). If there are many insignificant terms in the model (not counting those required to support hierarchy), then the elimination of these terms may improve the model.

Similarly, as shown in Table 3 for THC removal. The model F-value of 6.06 for 21 days treatment and 6.32 for the 56 days treatment implies the model is significant in terms of predicting TPH removal. Also, there is only a 3.07% and 2.81% probably chance that the obtained F-value from the 26 and 56 day model could occur due to noise. The model p-values are both less than the model reference p-value of 0.05 (5%) indicating that the model variable terms are significant in predicting the removal of THC. The following variable terms of C, AC, and B² are significant variable terms in the 21 days model while the B, B², and C² are significant variable terms in the 56 days model for THC removal. The other variable terms in both models are considered insignificant since their p-values are greater than 0.10 (10%). If there are many insignificant terms in the model (not counting those required to support hierarchy), then the elimination of these terms may improve the model.

Table 3 also shows the values of the coefficient estimate which is the intercept in an orthogonal design of the overall average response of all the runs for TPH and THC removal for days 21 and 56 models. The values as shown in Table 3 all indicate the entire runs response are significant.

**Figure 1.**
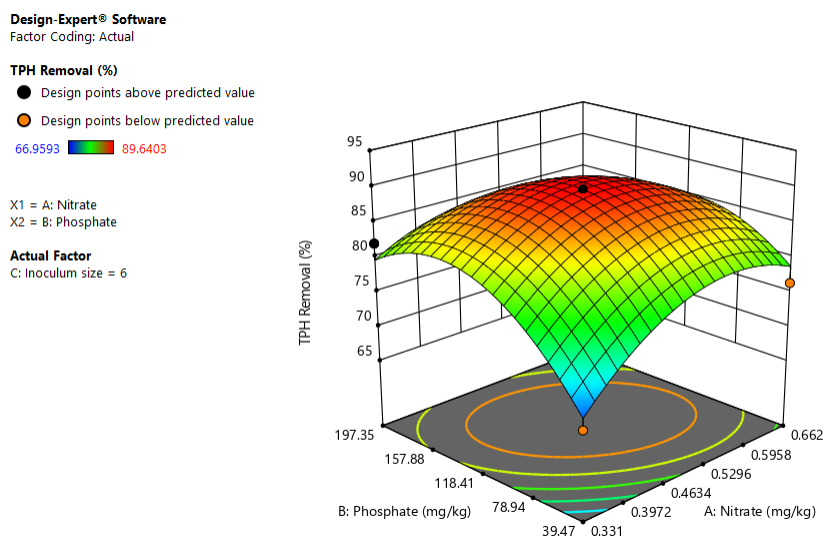
Surface 3D plot of total petroleum hydrocarbon (TPH) removal (%) as a function of phosphate and nitrate concentration at a 6% inoculum size after 21 days of cocktail treatment.

Figure 1 shows that an increase in both phosphate and nitrate concentration increases the TPH removal rate up to a certain point. Precisely TPH removal decreases at both the low and high concentration limit of phosphate and nitrate. But optimal TPH removal is around the mid-level leaning more toward the high concentration limit of both phosphate and nitrate.

**Figure 2.**
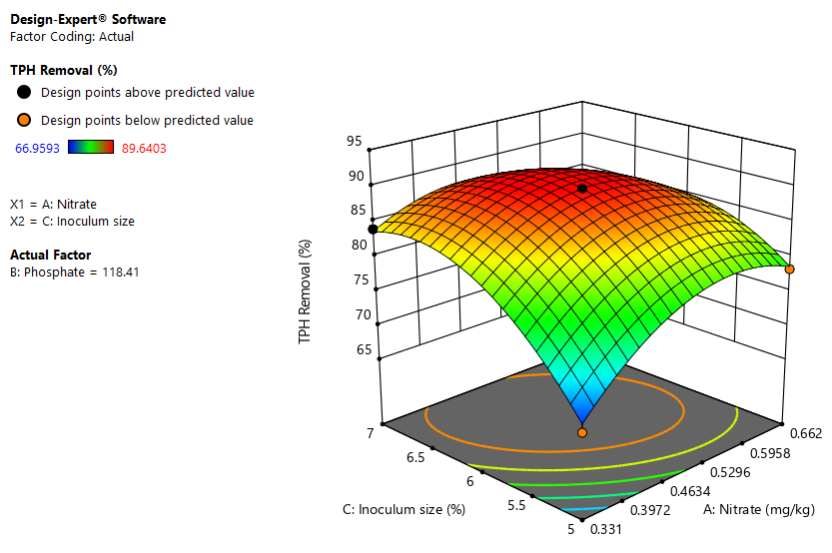
Surface 3D plot of total petroleum hydrocarbon (TPH) removal (%) as a function of inoculum size and nitrate concentration at constant phosphate concentration of 118.41 mg/kg after 21 days of cocktail treatment.

Figure 2 shows the interactive effects of inoculum size and nitrate on TPH removal at a constant phosphate concentration. It further shows that the THP removal rate would improve with an increase in the inoculum size. While TPH removal rate increases as the nitrate increase to a certain point, where further, increase in nitrate decrease the TPH removal rate. Optimal TPH removal is at high inoculum size and mid-level leaning toward high nitrate concentration.

**Figure 3.**
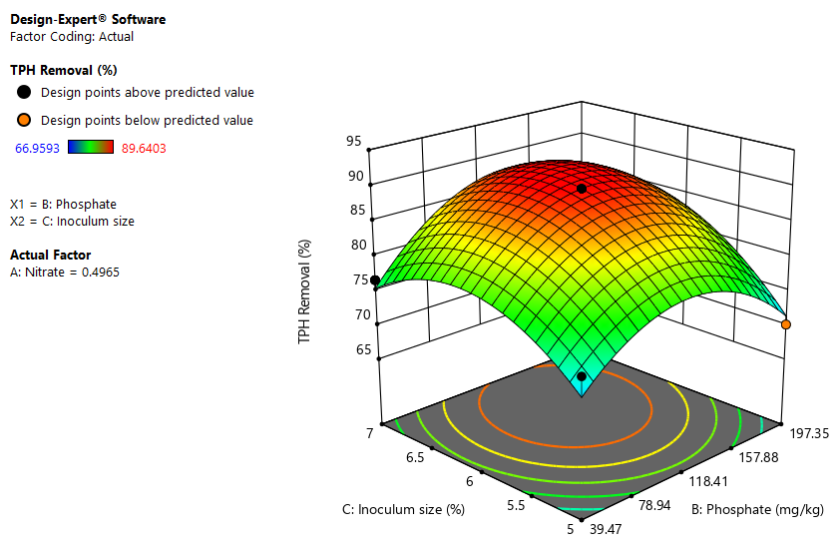
Surface 3D of total petroleum hydrocarbon (TPH) removal (%) as a function of inoculum size and phosphate concentration at a constant nitrate concentration of 0.497 mg/kg after 21 days of cocktail treatment.

Figure 3 shows the interactive effects of inoculum size and phosphate on TPH removal at a constant nitrate concentration. Both increases in inoculum size and phosphate increased TPH removal. However, at low phosphate concentration, high inoculum size does not result to increase TPH removal. Optimal TPH removal at constant nitrate is at high phosphate concentration and inoculum size.

**Figure 4.**
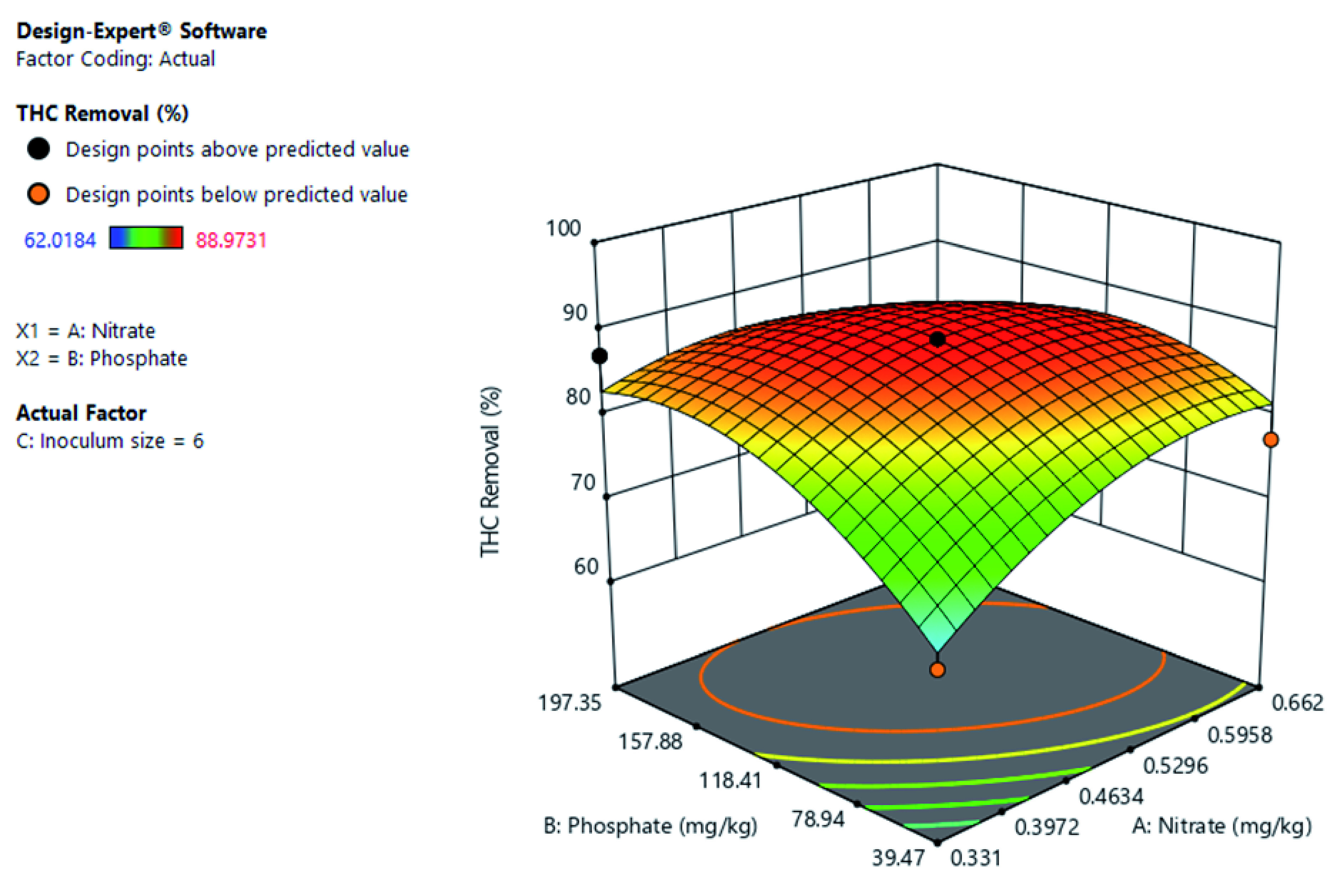
Surface 3D Plot of total hydrocarbon content (THC) Removal (%) as a function of Nitrate and Phosphate concentration at a constant 6% Inoculum size after 21 days of Cocktail treatment.

Figure 4 shows the interactive of nitrate and phosphate on THC removal at constant inoculum size. The increase in both nitrate and phosphate concentration increases THC removal. But the further increase of nitrate beyond certain points decreased THC removal.

**Figure 5.**
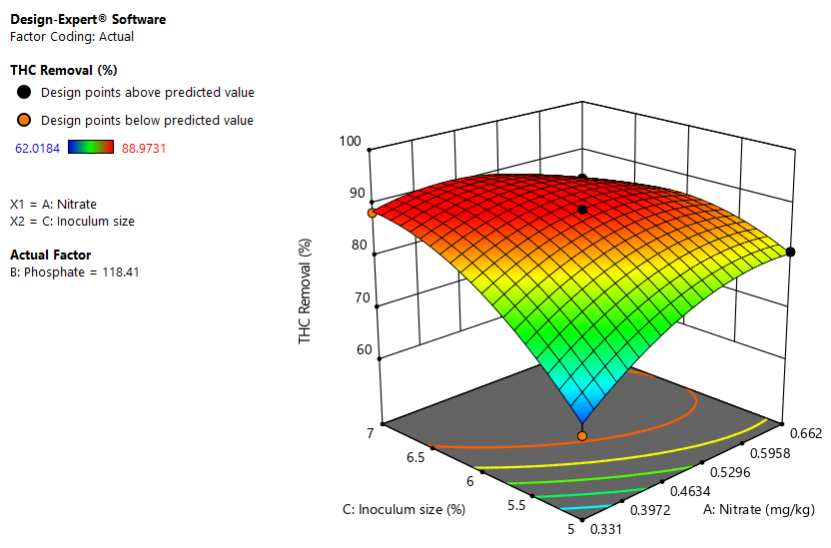
Surface 3D Plot of total hydrocarbon content (THC) Removal (%) as a function of Nitrate concentration and Inoculum size at a constant Phosphate concentration of 118.41 mg/kg after 21 days of Cocktail treatment.

Figure 5 shows the interactive effects of inoculum size and nitrate on THC removal at a constant phosphate concentration. It further shows that THC removal rate would improve with an increase in inoculum size. The THC removal rate would improve as nitrate increase to a certain point where further increase in nitrate decrease THC removal rate. Optimal THC removal is at high inoculum size and mid-level leaning toward high nitrate concentration.

**Figure 6.**
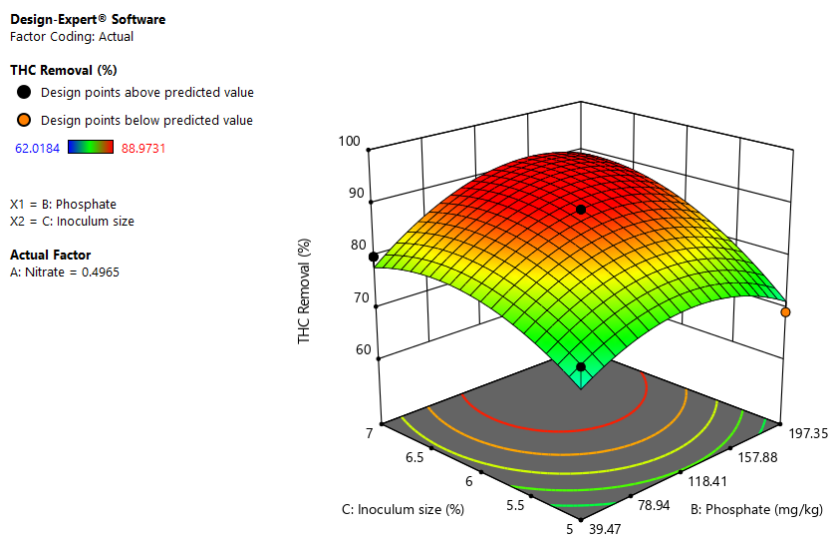
Surface 3D Plot of total hydrocarbon content (THC) Removal (%) as a function of Inoculum size and Phosphate Concentration at constant Nitrate concentration of 0.497 mg/kg after 21 days of Cocktail treatment.

Figure 6 shows the interactive effect of inoculum size and phosphate concentration at constant nitrate concentration. Increase in both inoculum size and phosphate concentration increased THC removal. Further shows that optimal THC removal at the high-level inoculum size and phosphate concentration.

**Figure 7.**
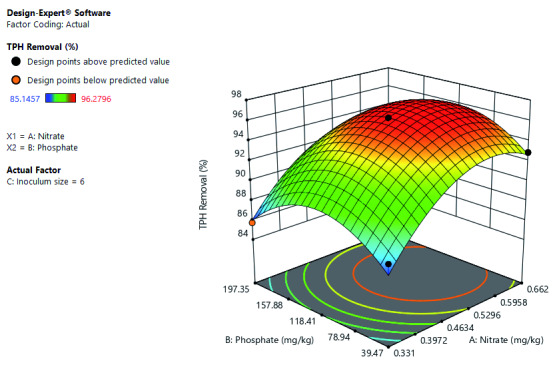
Surface 3D plot of Total Petroleum Hydrocarbon (TPH) Removal (%) as a function of Phosphate and Nitrate concentration at a 6% Inoculum size after 56 days of Cocktail treatment.

Figure 7 shows that the TPH removal rate improves with the increase in phosphate and nitrate at constant inoculum size. However, further, increase in phosphate to a certain level of concentration decrease the TPH removal rate. Optimal TPH removal occurs at a mid-level concentration of phosphate and between mid-level to high-level concentration of nitrate. It also depicts the interactive effects of both phosphate and nitrate on TPH removal.

**Figure 8.**
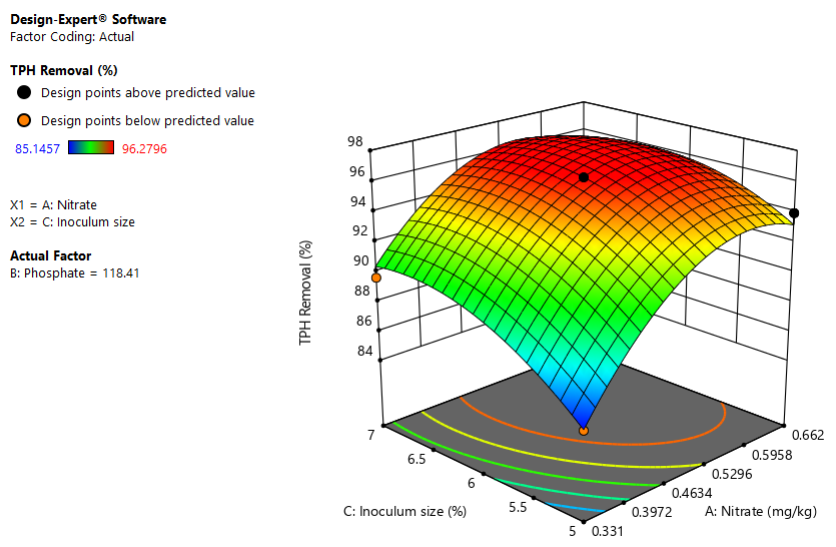
Surface 3D Plot of Total Petroleum Hydrocarbon (TPH) Removal (%) as a function of Inoculum size and Nitrate concentration at constant Phosphate concentration of 118.41 mg/kg after 56 days of Cocktail treatment.

Figure 8 shows the significance of the interactive effect of inoculum size and nitrate concentration at constant phosphate concentration. The increase in both inoculum size and nitrate concentration increased TPH removal. Further shows that optimal TPH removal at the high-level inoculum size and between mid-level to a high level of nitrate concentration.

**Figure 9.**
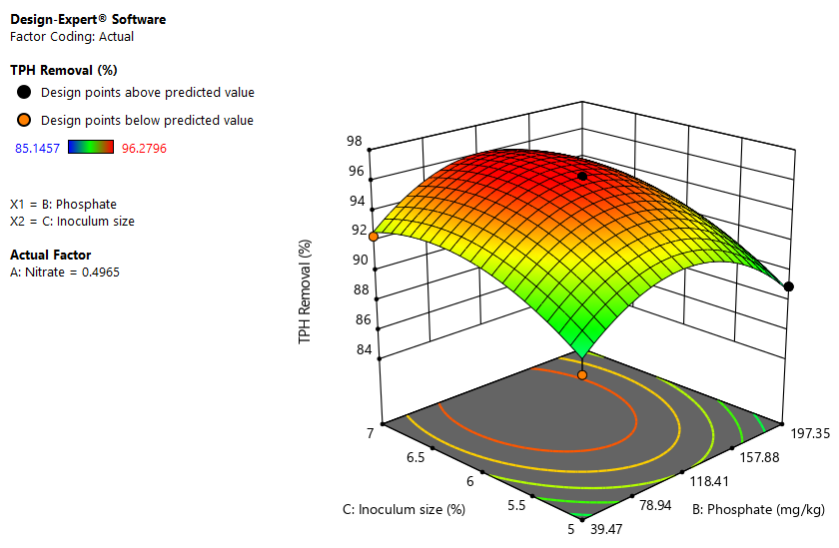
Surface 3D of Total Petroleum Hydrocarbon (TPH) Removal (%) as a function of Inoculum size and Phosphate concentration at a constant Nitrate concentration of 0.497 mg/kg after 56 days of Cocktail treatment.

Figure 9 shows that the TPH removal rate improves with the increase in phosphate and inoculum size at constant nitrate concentration. However, further, increase in phosphate to a certain level of concentration decrease the TPH removal rate. Optimal TPH removal occurs at a mid-level concentration of phosphate and between mid-level to high-level inoculum size. It also depicts the interactive effects of both phosphate and inoculum size on TPH removal.

**Figure 10.**
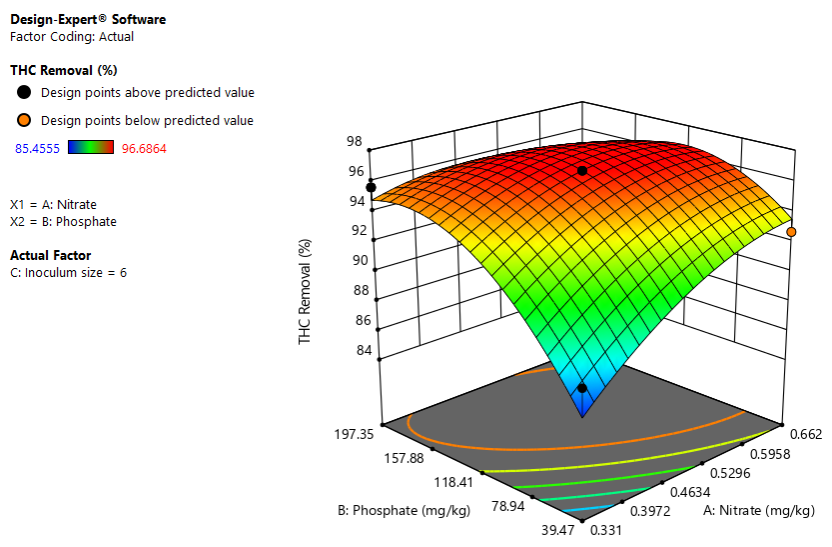
Surface 3D Plot of total hydrocarbon content (THC) Removal (%) as a function of Nitrate and Phosphate concentration at a constant 6% Inoculum size after 56 days of Cocktail treatment.

Figure 10 shows the interactive effects of phosphate and nitrate on the THC removal rate at a constant inoculum size. It further shows that THC removal increased with increase in phosphate. While THC removal increases as nitrate increases to a certain point. Further, the increase in nitrate towards high-level concentration decrease THC removal. Optimal THC removal is at high-level concentration of phosphate and mid-level leaning toward high nitrate concentration.

**Figure 11.**
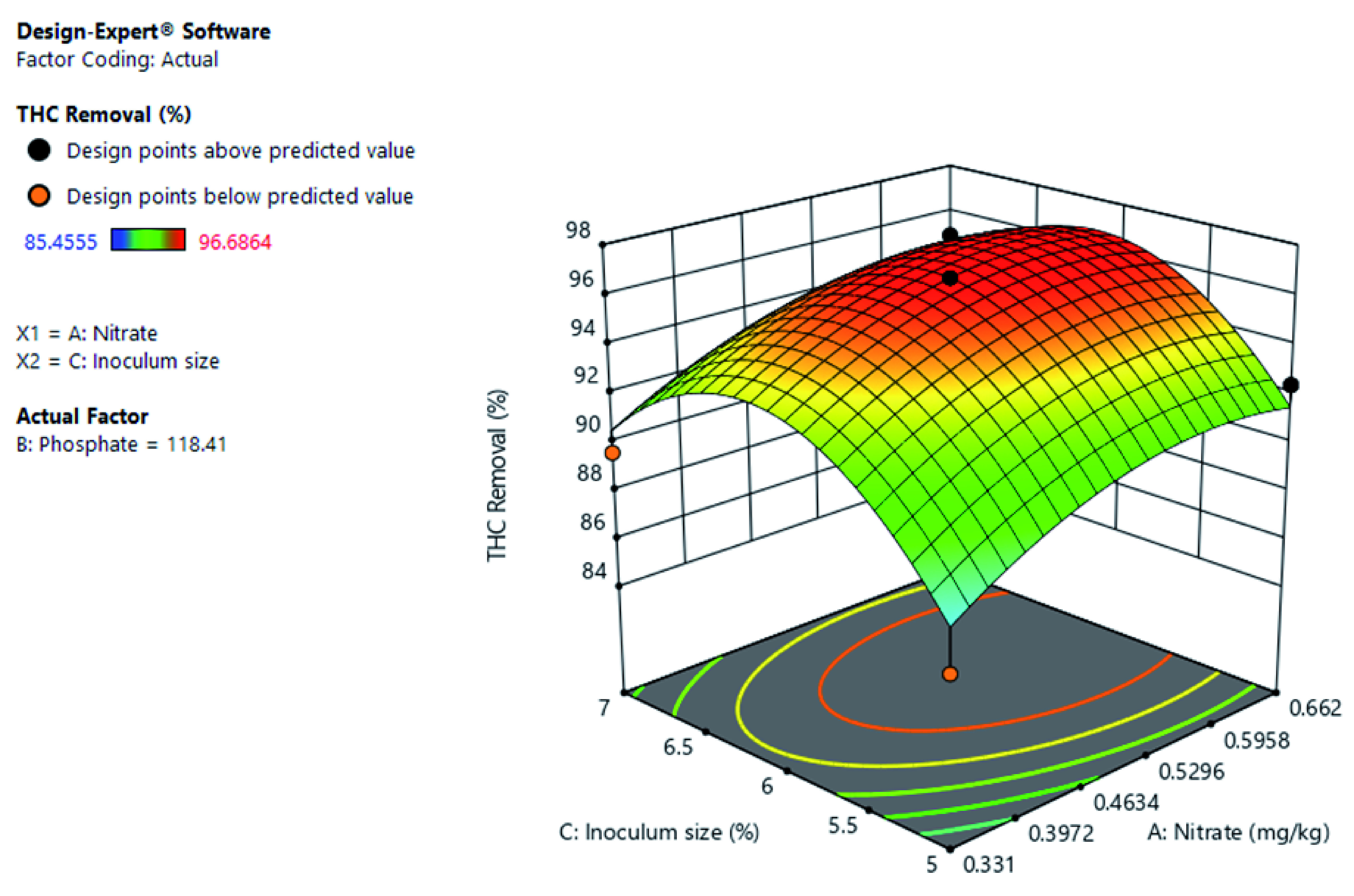
Surface 3D Plot of total hydrocarbon content (THC) Removal (%) as a function of Nitrate concentration and Inoculum size at a constant Phosphate concentration of 118.41 mg/kg after 56 days of Cocktail treatment.

Figure 11 shows the significance of the interactive effect of inoculum size and nitrate concentration at constant phosphate concentration. The increase in nitrate concentration increased the THC removal rate. While THC removal increased with inoculum size to a certain point. Further shows that optimal THC removal is at mid-level inoculum size and mid to high level of the phosphate concentration.

**Figure 12.**
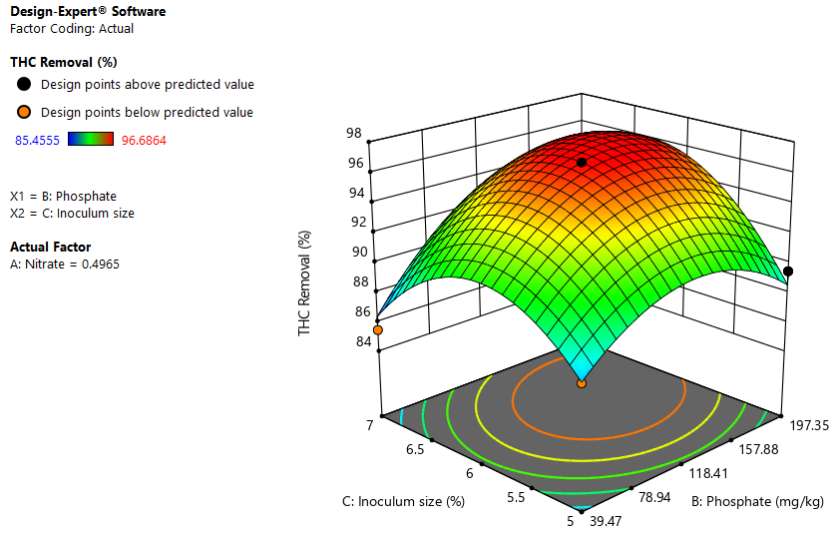
Surface 3D Plot of total hydrocarbon content (THC) Removal (%) as a function of Inoculum size and Phosphate Concentration at constant Nitrate concentration of 0.497 mg/kg after 56 days of Cocktail treatment.

Figure 12 shows the interactive effects of inoculum size and phosphate on THC removal at a constant nitrate concentration. Both increases in inoculum size and phosphate increased THC removal. However, at low phosphate concentration, high inoculum size does not result to increase THC removal. Optimal THC removal at constant nitrate is at high phosphate concentration and inoculum size.

## Discussion

The presence and applicability of rhizobacterial flora was previously reported by Kirkpatrick *et al.* (2008). Their study further documented the presence of highly functional bacterial flora and established the pattern and presence of highly degradative pathways for the sampled bacterial isolates. Their study, however, failed to apply effective flora to eco-recovery of a polluted matrix. This study shows the possibility of using a 6% (60ml) inoculum of the microbial consortia (which is less than 0.03 mg of the lyophilized consortia from a 100ml mixed culture) to remediate a significant expanse of impacted land, which is huge progress in the field as the small consortium was effective at remediation of up to 2 kg soil. Similarly, Orhorhoro *et al.* (2018) applied rhizobacteria established the preponderance of *Bacillus* sp. and *Pseudomonas* sp. as the most frequent in bacterial isolates associated with polluted environmental media with a high biodegradation rate.

Nutrient limitation in crude oil polluted media has been reported to have an adverse effect on both the physicochemical and microbiological qualities of the impacted soil ( Nwogu *et al.*, 2015). The changes in the soil could be extreme ranging from a total loss of soil fertility to the sterility of microbiota. The presence or absence of nutrients in the process of bioremediation have been associated with the increase of microbial population and moderation of nutrients and other physicochemical parameters of the soil. According to Meeboon *et al.* (2018) finding supporting the presence of nutrients as a key factor that may also affect the microbial population during a treatment process. The application of exogenous nutrients which attached to the soil fraction could serve as limiting nutrients through slow-release ( Khadem & Raiesi, 2017).

In the present study, 0.510 mg/kg (Corn steep liquor), phosphate 137.49 mg/kg and 6.4% inocula from biodegrading rhizobacteria were attained as optimal conditions for the design of bioremediation cocktail for treatment of hydrocarbon pollution at 9500mg/kg of contaminated soil, Box and Behnken approach was employed at p-value < 0.05 as summarized in Table 3. The model p-value was 0.009 at the F-stat value of 10.84 and was observed to be significant for the % removal of THC. Optimal loss of TPH at the 21 ^st^ day of monitoring was observed to be 89.64% and 96.28% on the 56 ^th^ day of the study ( Table 2 and Table 4), which fitted into a 2 ^nd^- order quadratic model as described in Table 3. This further implies that two or more variables interact significantly in the attainment of crude oil pollution removal and bioremediation of polluted soil. This account was in agreement with Ofoegbu (2015) whose findings validate the role of organic and inorganic fertilizers as substrates for limiting nutrients, for which their study yielded 71.80% and 63.54% removal in TPH level.

**Figure 13.**
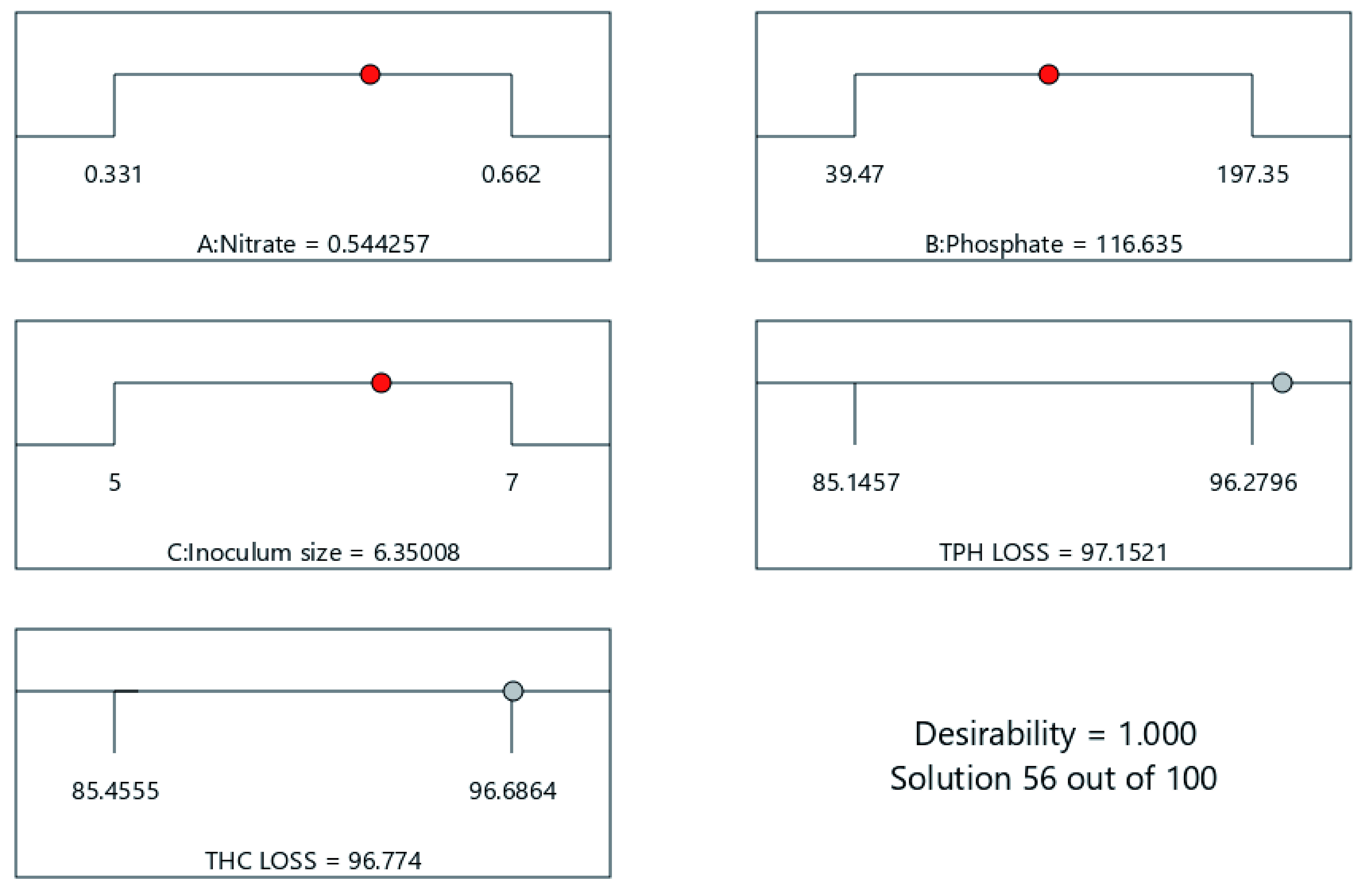
Optimization ramps showing response surface model predicted optimal cocktail formulation for 56 days of cocktail treatment.

**Figure 14.**
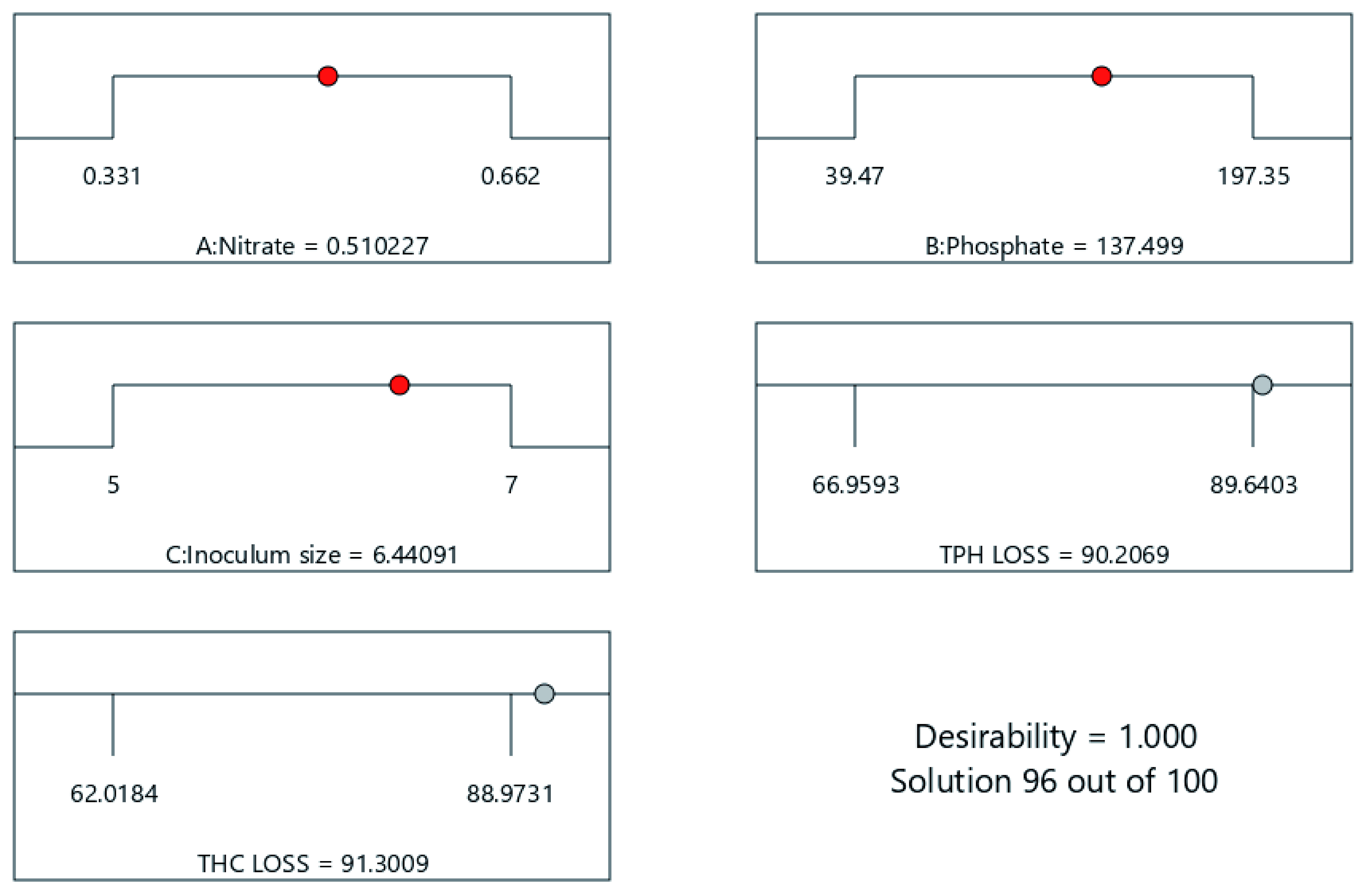
Optimization ramps showing response surface model predicted optimal cocktail formulation for 21 days of cocktail treatment.

The finding of this study is consistent with that Ofoegbu (2015), that the organic amendment of limiting nutrient source to polluted environmental matrices has resulted in improvement in both microbial activity and hydrocarbon contaminant removal. This suggests the applicability of Shelford’s law of the minimum to the application process of bioremediation of crude oil-polluted soil as suggested by Nwogu *et al.* (2015). The inaugural lecture by Abu (2017) called these nutrients that control the process and chemistry of biodegradation as ‘eco force’ as they principally influence the degree of removal of the contaminant of concerns. The need to optimize nutrients ( Figure 13 and Figure 14) in the formulation of bio-cocktails for eco-recovery has been harnessed in several peer-review articles with the unique desire to develop a nutrient formulation that meets specific needs in bioremediation application. This further asserts the development of cost-effective bioremediation material as it regards the development of process parameters to meet the growing needs for commercial field scale application.

## Conclusion

Optimization of bioremediation cocktails in the treatment of crude oil polluted environment was achieved. The combination of a system that harnesses bioaugmentation and biostimulation has been identified as a solution to the lingering environmental pollution. The use of agrowaste residues offers a sustainable path to the treatment of polluted environmental media. This study has established the feasibility of the application of a mathematical model in the development of an efficient bioremediation agent for the removal of contaminants. The removal of THC and TPH from the experimental samples suggests that a future that employs waste to wealth technology especially in the treatment and recovery of contaminated sites is possible and feasible.

## Data availability

### Underlying data

Open Science Framework: BIOREMEDIATION-COCKTAIL FOR ECO-RECOVERY OF IMPACTED ENVIRONMENT. https://doi.org/10.17605/OSF.IO/6ND92 ( Nwaichi, 2020).

This project contains the following underlying data:

- Agbaji Total Hydrocar Content (THC) results of polluted soil samples treated with biococktail composition formulated by the DX model.csv (Total hydrocarbon content of oil contaminated soil collected on day 1)
- Agbaji Total Hydrocar Content (THC) results of polluted soil samples treated with biococktail composition formulated by the DX model day 7.csv (Total hydrocarbon content of oil contaminated soil collected on day 7)
- Agbaji Total Hydrocar Content (THC) results of polluted soil samples treated with biococktail composition formulated by the DX model day 14.csv (Total hydrocarbon content of oil contaminated soil collected on day 14)
- Agbaji Total Hydrocar Content (THC) results of polluted soil samples treated with biococktail composition formulated by the DX model day 21.csv (Total hydrocarbon content of oil contaminated soil collected on day 21)
- Agbaji Total Hydrocar Content (THC) results of polluted soil samples treated with biococktail composition formulated by the DX model day 56.csv (Total hydrocarbon content of oil contaminated soil collected on day 56)
- For Agbaji Actual data conversion to input data for Design Expert modelling of cocktail composition and application.csv (Data input for modelling)
- For Agbaji Bioremediation Cocktail composition and THP and THC initial concentration in polluted soil for treatment.csv (Baseline measurements for bioremediation cocktail, THP and THC)
- For Agbaji Total Petroleum Hydrocarbon (TPH) taken from polluted baseline soil sample not treated with biococktail on Day1.csv (raw data of Gas Chromatographic (GC) readings for total petroleum hydrocarbons (TPH) collected on day 1)
- For Agbaji TPH for Day 1_21_56 taken from HC polluted soil treated with biococktail formulated from Sample 1 DX composition.csv (raw data of Gas Chromatographic (GC) readings for total petroleum hydrocarbons (TPH) collected on days 1, 21 and 56 of the bioremediation cocktail application to hydrocarbon contaminated soil samples. Sample 1)
- For Agbaji TPH for Day 1_21_56 taken from HC polluted soil treated with biococktail formulated from Sample 2 DX composition.csv (raw data of Gas Chromatographic (GC) readings for total petroleum hydrocarbons (TPH) collected on days 1, 21 and 56 of the bioremediation cocktail application to hydrocarbon contaminated soil samples. Sample 2)
- For Agbaji TPH for Day 1_21_56 taken from HC polluted soil treated with biococktail formulated from Sample 3 DX composition.csv (raw data of Gas Chromatographic (GC) readings for total petroleum hydrocarbons (TPH) collected on days 1, 21 and 56 of the bioremediation cocktail application to hydrocarbon contaminated soil samples. Sample 3)
- For Agbaji TPH for Day 1_21_56 taken from HC polluted soil treated with biococktail formulated from Sample 4 DX composition.csv (raw data of Gas Chromatographic (GC) readings for total petroleum hydrocarbons (TPH) collected on days 1, 21 and 56 of the bioremediation cocktail application to hydrocarbon contaminated soil samples. Sample 4)
- For Agbaji TPH for Day 1_21_56 taken from HC polluted soil treated with biococktail formulated from Sample 5 DX composition.csv (raw data of Gas Chromatographic (GC) readings for total petroleum hydrocarbons (TPH) collected on days 1, 21 and 56 of the bioremediation cocktail application to hydrocarbon contaminated soil samples. Sample 5)
- For Agbaji TPH for Day 1_21_56 taken from HC polluted soil treated with biococktail formulated from Sample 6 DX composition.csv (raw data of Gas Chromatographic (GC) readings for total petroleum hydrocarbons (TPH) collected on days 1, 21 and 56 of the bioremediation cocktail application to hydrocarbon contaminated soil samples. Sample 6)
- For Agbaji TPH for Day 1_21_56 taken from HC polluted soil treated with biococktail formulated from Sample 7 DX composition.csv (raw data of Gas Chromatographic (GC) readings for total petroleum hydrocarbons (TPH) collected on days 1, 21 and 56 of the bioremediation cocktail application to hydrocarbon contaminated soil samples. Sample 7)
- For Agbaji TPH for Day 1_21_56 taken from HC polluted soil treated with biococktail formulated from Sample 8 DX composition.csv (raw data of Gas Chromatographic (GC) readings for total petroleum hydrocarbons (TPH) collected on days 1, 21 and 56 of the bioremediation cocktail application to hydrocarbon contaminated soil samples. Sample 8)
- For Agbaji TPH for Day 1_21_56 taken from HC polluted soil treated with biococktail formulated from Sample 9 DX composition.csv (raw data of Gas Chromatographic (GC) readings for total petroleum hydrocarbons (TPH) collected on days 1, 21 and 56 of the bioremediation cocktail application to hydrocarbon contaminated soil samples. Sample 9)
- For Agbaji TPH for Day 1_21_56 taken from HC polluted soil treated with biococktail formulated from Sample 10 DX composition.csv (raw data of Gas Chromatographic (GC) readings for total petroleum hydrocarbons (TPH) collected on days 1, 21 and 56 of the bioremediation cocktail application to hydrocarbon contaminated soil samples. Sample 10)
- For Agbaji TPH for Day 1_21_56 taken from HC polluted soil treated with biococktail formulated from Sample 11 DX composition.csv (raw data of Gas Chromatographic (GC) readings for total petroleum hydrocarbons (TPH) collected on days 1, 21 and 56 of the bioremediation cocktail application to hydrocarbon contaminated soil samples. Sample 11)
- For Agbaji TPH for Day 1_21_56 taken from HC poluted soil treated with biococktail formulated from Sample 12 DX composition.csv (raw data of Gas Chromatographic (GC) readings for total petroleum hydrocarbons (TPH) collected on days 1, 21 and 56 of the bioremediation cocktail application to hydrocarbon contaminated soil samples. Sample 12)
- For Agbaji TPH for Day 1_21_56 taken from HC poluted soil treated with biococktail formulated from Sample 13 DX composition.csv (raw data of Gas Chromatographic (GC) readings for total petroleum hydrocarbons (TPH) collected on days 1, 21 and 56 of the bioremediation cocktail application to hydrocarbon contaminated soil samples. Sample 13)
- For Degradation Agbaji Day 21 and Day 56 Bioremediation cocktail composition as model by DX and their application response.csv

Data are available under the terms of the Creative Commons Zero "No rights reserved" data waiver (CC0 1.0 Public domain dedication).
