# Supplementary material for: Optimization of bioremediation-cocktail for application in the eco-recovery of crude oil polluted soil
Source: AAS Open Res. 2020 Apr 22;3:7. [Version 1] doi: 10.12688/aasopenres.13028.1 (PMC7551562; doi:10.12688/aasopenres.13028.1)
Supplement: Peer review history

# Peer review: Reviewer response for version 1

**DOI:** 10.21956/aasopenres.14119.r27836

**Published:** 2020-10-12

**Authors:** Mojisola Owoseni

**Affiliations:** 1 Federal University of Lafia, Lafia, Nigeria

Comments:

- The study addressed the use of agrowastes to support the use of high thoroughput rhizobacteria in the formulation of a bioremediation cocktail for reclamation of oil contaminated soils in Nigeria.
- The Introduction is well written with sufficient references to support the background of the study.

 

 Methodology:

- The subtitle “Microorganism” can be rephrased as “Microbial processing”.
- Some statements are disjointed and need to be rephrased.
- The physiochemical conditions in the bioremediation cocktail should have been taken on day zero, 7, 21 and 56 when the soil sample was taken out for measurement of TPH and THC. This will enable reproducibility and replicability.

 

 Discussions:

- The authors should provide more details to cover discussion of results. Results should be compared to other literature.

Is the work clearly and accurately presented and does it cite the current literature?

Yes

If applicable, is the statistical analysis and its interpretation appropriate?

Yes

Are all the source data underlying the results available to ensure full reproducibility?

Yes

Is the study design appropriate and is the work technically sound?

Yes

Are the conclusions drawn adequately supported by the results?

Yes

Are sufficient details of methods and analysis provided to allow replication by others?

Partly

Reviewer Expertise:

Environmental Microbiology

I confirm that I have read this submission and believe that I have an appropriate level of expertise to confirm that it is of an acceptable scientific standard.

# Peer review: Reviewer response for version 1

**DOI:** 10.21956/aasopenres.14119.r27464

**Published:** 2020-05-28

**Authors:** Farooq Sher

**Affiliations:** 1 School of Mechanical, Aerospace and Automotive Engineering, Faculty of Engineering, Environmental and Computing, Coventry University, Coventry, UK

**Major comments:**

 In this study, the authors have studied four hydrocarbon-degrading bacterial strains which were pre-selected from mined rhizobacterial isolates in aged crude oil-contaminated soil. Agro waste residues of poultry-droppings, corn chaff, and plantain peel were selected among others for their ability to support high biomass of selected bacterial strains. This study has shown the need to harness the abundant agro-waste nutrients in supporting high throughput rhizobacteria in the formulation of a bioremediation agent suitable for use in the reclamation of oil spill sites in the Niger Delta oil-producing region. English language used in the manuscript needs improvements as there are many punctuation and grammatical mistakes present throughout the manuscript. Some sentences need more clarity and better construction. Some of the figures need revision and more transparency, special focus is required in labelling the figures and tables. The authors are advised to addressing the following comments to enhance the quality of the manuscript.

**Introduction:**

 The introduction needs to be more strengthened with sufficient background information and a possible wide future application of this technology. The authors are also advised to use short, concise sentences to get the idea at once. It is also recommended to include some more comprehensive details about what the authors intended to do in a clear way and the possible future application.

**Methods/experimental section:**

 In the experimental section, the authors need to specify what authors have studied related to this work, what they intend to do and what was the need of this study clearly. The experimental section is quite standard and appropriate as it gives detailed information under appropriate headings and subheadings but needs some focus on describing the effect of process conditional changes.

**Results and discussions:**

 This section requires more in-depth discussion and special keenness as it arises a lot of questions regarding the effect of temperature, pressure and other parameters changes. The authors should consider sentence structural issues. The authors are advised to critically address their results with the previously published literature. Also, cross-check results and also narrate the limitations of the study. The authors are advised to add some key findings of the study by providing detailed keen analysis, justify with reference from the literature.

**Conclusions:**

 The conclusion needs to be more elaborative with strong reasoning at some points as conclusions only talk about some studied parameters. The authors are advised to write the conclusions in a comprehensive way including key values from the results, suitability of applied method, limitation, suggestions for further research and scope of this technology clearly. They need to give a strong argument and appropriate supporting evidence in favour of their present work.

Is the work clearly and accurately presented and does it cite the current literature?

No

If applicable, is the statistical analysis and its interpretation appropriate?

Yes

Are all the source data underlying the results available to ensure full reproducibility?

Partly

Is the study design appropriate and is the work technically sound?

Yes

Are the conclusions drawn adequately supported by the results?

Partly

Are sufficient details of methods and analysis provided to allow replication by others?

Partly

Reviewer Expertise:

Chemical Engineering

I confirm that I have read this submission and believe that I have an appropriate level of expertise to confirm that it is of an acceptable scientific standard.

# Response

**Published:** 2020-05-31

**Authors:** Eucharia Nwaichi

**Affiliations:** University of Port Harcourt, Nigeria

Dear Reviewer,

Thank you for your contributions to our work. Modifications in line with your suggestions are done in red. Additionally, under the headings, **Consideration and Deductions of Agrowaste Nutrient Concentration from Proximate Analysis Results, and Cocktail Formulation, the experimental's intent were declared.**

Temperature and pressure were not factors considered here and those of nutrients were highlighted in the second paragraph under 'Discussion'. 

Limitations have been added and a recent review was added.

Please see the attached file here.
